# Circular of American Medical Association

**Published:** 1874-05-01

**Authors:** Wm. B. Atkinson

**Affiliations:** Permanent Secretary; Philadelphia


### AMERICAN MEDICAL ASSOCIATION

PHILADELPHIA, 1400 PINE ST., S. W. COR. BROAD.

THE Twenty-fifth Annual Session will be held in the city of Detroit, Mich., on Tuesday, June 2d, 1874, at 11 A.M.

"The Chairman of the several sections shall prepare and read, in the general sessions of the Association, papers on the advances and discoveries of the past year in the branches of science included in their respective sections. * * * "—BY-LAWS, Art. II., Sect. 4.

SECTIONS.

*Practice of Medicine, Materia Medica, and Physiology.*—Dr. N. S. Davis, Chicago, Ill., Chairman; Dr. George E. Frothingham, Ann Arbor, Mich., Secy.

*Obstetrics, and Diseases of Women and Children.*—Dr. Theophilus Parvin, Indianapolis, Ind., Chairman; Dr. Montrose A. Pallen, St. Louis, Mo., Sec'y.

*Surgery and Anatomy.*—Dr. Sam'l D. Gross, Philadelphia, Pa., Chairman; Dr. Alonzo Garcelon, Lewiston, Me., Sec'y.

*Medical Jurisprudence, Chemistry, and Psychology.*—Dr. A. N. Talley, Columbia, S. C., Chairman; Dr. E. Lloyd Howard, Baltimore, Md., Sec'y.

*State Medicine and Public Hygiene.*—Dr. A. Nelson Bell, Brooklyn, N. Y., Chairman; Dr. A. B. Stuart, Winona, Minn., Secretary.

"Papers appropriate to the several sections, in order to secure consideration and action, must be sent to the Secretary of the appropriate section at least one month before the meeting which is to act upon them. It shall be the duty of the Secretary to whom such papers are sent to examine them with care, and, with the advice of the Chairman of his section to determine the time and order of their presentation, and give due notice of the same. * * * "—BY-LAWS, Art. II., Sect. 5.

The following Committees are expected to report:

*On the Cultivation of the Cinchona Tree.*—Dr. L. J. Deal, Pennsylvania, Chairman.

*On the Treatment of Fractures.*—Dr. Lewis A. Sayre, of New York, Chairman.

*On Gynæcology.*—Dr. M. A. Pallen, Missouri, Chairman.

*On some Diseases Peculiar to Colorado.*—Dr. John Elsner, Colorado, Chairman.

*On Rank of Medical Corps of the Army.*—Dr. J. M. Keller, Kentucky, Chairman.

*On Prize Essays.*—Dr. G. K. Johnson, Michigan, Chairman.

*On the Progress of Otology.*—Dr. D. B. St. John Roosa, New York, Chairman.

*On American as Compared with Foreign Winter Cures.*—Dr. H. R. Storer, Massachusetts, Chairman.

*On Railroad Injuries.*—Dr. W. F. Peck, Iowa, Chairman.

*On the Therapeutics of Ammonia.*—Dr. P. J. Farnsworth, Iowa, Chairman.

*On the Relations of Physiology to the Practice of Medicine.*—Dr. E. W. Gray, Illinois, Chairman.

*On Puerperal Fever.*—Dr. W. O. Smith, Kentucky, Chairman.

*On the Legal Relations of Moral Insanity.*—Dr. E. Lloyd Howard, Maryland, Chairman.

The following amendments to the Plan of Organization are to be acted upon:

*By Dr. N. S. Davis, Illinois:* Strike out the second paragraph of Art. II., and insert the following: "The delegates shall receive their appointment from permanently organized State Medical Societies, and such County and District Medical Societies as are recognized by representation in their respective State Societies, and from the Medical Department of the Army and Navy of the United States." Also, strike out the fourth paragraph of same Article, and insert, "Each State, County, and District Medical Society, entitled to representation, shall have the privileges of sending to the Association one delegate for every ten of its regular resident members, and one for every additional fraction of more than half that number.

"The Medical Staffs of the Army and Navy shall be entitled to four delegates each."

*By Dr. B. Pineo, of Massachusetts:* Art. II., second paragraph after "Army and Navy," insert "and the Marine Hospital Service of the United States." By-Laws, Sect. 6, after "the chiefs of the bureaus of the Army and Navy," insert "and the supervising surgeon of the United States Marine Hospital Service."

*By Dr. E. L. Howard, of Maryland:* Art. IV., strike out second clause of first paragraph, and insert, "They shall be nominated by the Judicial Council, and shall be elected by vote on a general ticket."

*By Dr. A. S. Maxwell, of Iowa.*—"*Resolved,* That in view of the many and important duties imposed upon the Nominating Committee, the Medical Society of each State and Territory that elects delegates be requested, when selecting delegates, to nominate one member of such delegation as their member of the Nominating Committee; and also designate the mode of filling vacancies."

*By Dr. A. M. Pollock, of Pennsylvania:* Art. VI., first paragraph, strike out the word "five" and insert "ten." By-Laws, Art. V., first paragraph, strike out "five" and insert "ten."

*Secretaries of all Medical organizations that have adopted the Code of Ethics are respectfully requested to forward to the undersigned a complete list of their Officers, with their Postoffice addresses, and the number of their members in good standing. This is the only guide for the Committee of Arrangements in determining as to the reception of delegates.*

*It will also enable the Permanent Secretary to present a correct report of the Medical organizations in fellowship with the Association.*

WM. B. ATKINSON, M.D.,

*Permanent Secretary.*

